# Obituary Notice of Dr. Andrew Combe

**Published:** 1847-11

**Authors:** 


*Doctor Andrew Combe.*—Of the death of Dr. Combe, our readers have been apprised through the public papers. On us devolves the duty of indicating the special merits of our deceased brother, and the loss to the profession by his death. Here, if any intelligent reader were at our side, he might interrupt us by remarking on the signal services rendered to mankind by the writings of Dr. Combe, and allege that still, in a greater degree, must his loss be felt by the members, generally, of every community, than even by his professional brethren. Literally, and in its full meaning, may it be said, that man, woman and child, are under the strongest obligations to this popular writer, for his singularly lucid and practical teaching of hygiene, in connexion with physiology, as evinced in his different works, of which we shall soon speak under their respective titles. In another point of view, the labors of Dr. Combe furnish a lesson of encouragement to those who, owing to failing health, might be deterred from prosecuting their literary and professional labors, and of reproof to others who plead every slight indisposition and mental depression as excuses for not discharging their duties in these respects. He became a victim to the inroads of pulmonary consumption soon after the age of manhood; but yet he continued to be the zealous student, and at intervals the industrious and successful writer, during the whole of the period between the date of his first attack of disease, in 1820, and that of his death, on the 9th of August, 1847, a little before he had attained his fiftieth year.

Dr. Combe was born in Edinbrrgh on the 27th of October, 1797, being the fifteenth child, and seventh son, of parents whose entire progeny numbered seventeen. Having gone through the usual course of instruction at the High School, he was bound apprentice, in conformity with the usage in Great Britain, to the late Henry Johnston, Esq., surgeon in Edinburgh, and in 1817 he had acquired an amount of knowledge which entitled him to be licensed as a surgeon. With the view of farther qualifying himself for medical practice, he next repaired to Paris, where two years were laboriously spent in attending on the hospitals and listening to the instruction so prodigally given out in the lectures of the many professional celebrities of that capital. In 1823 he began to practice in Edinburgh, and about two years later took there his medical degree. Success attended his professional labors, marked as they were by sagacity, kindliness and conscientiousness; and he was in the course of a few years in the enjoyment of a flourishing practice. But a return of symptoms of pulmonary disease obliged him, in 1831, to proceed to Italy, where he had been once before from a similar cause. He was, however, able to pass the winter of 1832–3 in Scotland, and in the latter year to resume his practice. "In 1836 he was honored with the appointment of Physician in Ordinary to the King and Queen of the Belgians, and for several months attended the royal family in Brussels; but the climate proving unfavorable to him, an alarming return of his pulmonary symptoms abruptly sent him back to recruit his health in his native land. Subsequently he continued to act as consulting physician to their Majesties, and occasionally paid them a visit. About six or seven years ago he was appointed one of the Physicians Extraordinary to the Queen in Scotland, and afterwards one of her majesty's physicians in Ordinary in this part of the united kingdom. He was, also, a fellow of the Royal College of Physicians of Edinburgh, and a corresponding member of the Imperial and Royal Society of Physicians of Vienna."

The works by which Dr. Combe is best known to the public, are—"The Principles of Physiology applied to the Preservation of Health, and to the Improvements of Physical and Mental Education," of which twelve editions have been called for since its first appearance in 1834; "The Physiology of Digestion, considered with Relation to the Principles of Dietetics," originally published in 1836, and now in the seventh edition; and "A Treatise on the Physiological and Moral Management of Infancy, for the use of Parents," of which the first edition came out in 1840, and the sixth or People's edition, in June of the present year.

These works have been republished in the United States, and the first and third mentioned have gone through many editions. This last was edited by Dr. Bell in a manner that gave much satisfaction to the author. As it is the fashion, just now, with some of our home critics, and particularly with those who are quite innocent of any literary attempt themselves, to speak in terms of disparagement of American editions of English works, we shall quote the concluding paragraph of the introduction to the last edition of the "Treatise on Infancy:"—"Here I cannot resist the opportunity of expressing my grateful acknowledgements to Dr. John Bell, of Philadelphia, for the time and trouble which, amidst many pressing avocations, he so kindly and disinterestedly bestowed, not merely in superintending the republication of this work in the U. S. but in enriching it with many valuable notes, for the purpose of adapting it more completely to the domestic habits and wants of the Transatlantic public. To Dr. Blicker, of

Copenhagen, I am also indebted for its appearance in a Danish translation."

The aim of Dr. Combe, in preparing these works, is well expressed in the following sentences, by the author himself. "In teaching dietetic rules and hygienic observances, therefore, the precepts delivered should be connected with and supported by constant references to the Physiological laws from which they are deduced. Thus viewed they come before the mind of the reader as the mandates of the Creator; and experience will soon prove, that by his appointment, health and enjoyment flow from obedience, and sickness and suffering from neglect and infringement of them." That he was entirely successful in his estimate, both of the importance of the subjects, and of the manner in which they are best taught, is shown by, not merely the wide circulation, but the careful study of his different treatises.

Dr. Combe and his elder brother, Mr. George Combe, with whom in this country he is often confounded, were among the leading members of the Edinburgh Phrenological Society, instituted in 1820. He contributed two essays to the volume of Transactions, published by that body in 1824, and subsequently wrote many valuable papers in the *Phrenological Journal*, which was commenced in 1823, and now extends to twenty volumes. In 1831, Dr. Combe published "Observations on Mental Derangement; being an application of the Principles of Phrenology to the Elucidation of the Causes, Symptoms, Nature, and Treatment of Insanity." This work has long been out of print; the infirm health of the author having, as he tells us, prevented him from devoting that attention to the treatment of insanity, and, consequently, of "doing that justice to the subject, which its latter progress and inherent importance imperatively demand." In the beginning of 1846, his strong conviction of the importance of phrenology to medical men, induced him to write, at the penalty of considerable fatigue, an "Address to the students of Anderson's University, Glasgow, at the opening of Dr. Weir's first course of lectures on Phrenology."* It was delivered to a crowded audience by his brother, (Mr. George Combe,) and subsequently appeared as a pamphlet. He also contributed several articles to the *British and Foreign Medical Review*, and was an occasional writer on medical and sanitary subjects in the columns

*Printed in the London Lancet, and in the Bulletin of Medical Sciences, edited by Dr. Bell.

of the *Scotsman*, a paper conducted with great ability and exerting no little political influence in North Britain.

From the biographical sketch of Dr. Combe, which appeared in the Scotsman of August 21, we extract the following characteristic traits of the deceased, in the accuracy of which we have full reliance. For ourselves we cannot boast of the advantage of much personal intercourse with Dr. Combe; but, even from the slight acquaintance with him which his few days' visit to Philadelphia, in the early part of the last summer, allowed us to make, we are prepared to adopt the opinions of his friend in the paper just referred to.

"The disease of Dr. Combe, will have taken no one who knew him by surprise, for he was for many years in that condition, with the loss entirely of one lung, which makes life a greater miracle than death; but it will not on this account be the less deplored, either as causing a blank in the circle of private friendship, or as the signification of a public loss. Dr. Combe belonged to that rare class of physicians who present professional knowledge in connection with the powers of a philosophical intellect, and yet, in practical matters, appear constantly under the guidance of a rich natural sagacity. All of his works are marked by a peculiar earnestness, lucidity and simplicity, characteristic of their author; they present hygienic principles with a clearness for which we have no parallel in medical literature. To this must be ascribed much of the extraordinary success they have met with, and on this quality undoubtedly rests no small share of their universally acknowledged utility. Those, however, who look below the surface will not fail to trace a deep philosophical spirit, as pervading these works, something arising from a perfect apprehension of, and a perfect allegiance to the natural rule of God in our being. It has been a guidance, one would almost say an inspiration, of the author, without ever carrying him for a moment where ordinary readers could not follow him. Here we think is the true though latent strength of Dr. Combe's popular writings, and that which will probably give them a long, enduring pre-eminence in their particular department. We always feel, in reading them, that we are listening to one of those whom nature has appointed to expound and declare her mysteries for the edification of her multitudinous family. In his own section of her priesthood, certainly few have stood in his grade, fewer still become his superiors.

"The personal character and private life of Dr. Combe formed a beautiful and harmonious commentary upon his writings. In the bosom of his family and the limited social circle to which his weakly health confined him, he was the same benignant and gentle being whom the world finds addressing it in these campositions. The same clear sagacious intelligence, the same entire right-mindedness, shone in his conversation. An answer to any query put to him, whether respecting profession or miscellaneous matters, was precisely like a passage of one of his books, earnest, direct and conclusive. Whatever measures he called upon others to do or to avoid, that he did, and that he avoided, in his own course of life; for doctrine, with him, was not to be treated as external to himself, but as the expression of a system of divine appointment, of which he was a part. To his rigid though unostentatious adherence to the natural laws which he explained, it was owing, that he sustained himself for many years in a certain measure of health and exemption from suffering, while laboring under the consumptive tendency which finally has cut short his career. On this point, there is the more reason to speak emphatically, when we reflect that the years thus redeemed from the grave were employed in that which will yet save many from premature death, as if it had been his aim to show the value of even the smallest remains of life and strength, and thus advance one of the principles dearest to humanity. It was not, however, in any of these respects that the character af Dr. Combe made its best impression, but in his perfect geniality and simplicity, and the untiring energy of his practical benevolence. Here resided the true charm of his nature, and that which made him the beloved of all who knew him. No irritability attended his infirm health; no jealousy did he feel regarding those whom superior strength enabled to outstrip him in the professional race. Kindly and cordially to all, he did not seem to feel as if he could have an enemy, and therefore we believe he never had one. It might almost be said that he was too gentle and unobtrusive—and so his friends, perhaps, would have thought him, had it not, on the other hand, appeared as the most befitting character of one who, they all knew, was not to be long spared to them, and on whom the hues of a brighter and more angelic being seemed already to be shed."

In a letter from a near relative of Dr. Combe, announcing the death of the latter, addressed to Dr. Bell, it is stated that Dr. C., after going to bed on the evening of the 2d of August, in his usual state of health, was seized with diarrhœa, under which, after ineffectual attempts to check it, he sank on the 9th of the same month. He had very little suffering, and his mind continued calm and cheerful to the last. Frequently, he expressed his thankfulness that he was permitted to depart so easily. The immediate cause of his death was ascertained, in *post mortem* examination, to be a "chronic disease of the bowels, terminating in ulceration." "The left lung was wasted away and completely useless; the right nearly, or altogether entire." For years past Dr. Combe knew that his left lung was gone; his chest on that side had sunk in. We shall probably yet receive a more detailed account of the structural changes from Dr. John Scott, who was his professional attendant and friend.

